# Identification of Phytoconstituents in *Leea indica* (Burm. F.) Merr. Leaves by High Performance Liquid Chromatography Micro Time-of-Flight Mass Spectrometry

**DOI:** 10.3390/molecules24040714

**Published:** 2019-02-16

**Authors:** Deepika Singh, Yin-Yin Siew, Teck-Ian Chong, Hui-Chuing Yew, Samuel Shan-Wei Ho, Claire Sophie En-Shen Lim, Wei-Xun Tan, Soek-Ying Neo, Hwee-Ling Koh

**Affiliations:** Department of Pharmacy, Faculty of Science, National University of Singapore, 18 Science Drive 4, Singapore 117543, Singapore; yindividual@hotmail.com (Y.-Y.S.); TI_Chong90@gmail.com (T.-I.C.); youyiting1979@yahoo.com.sg (H.-C.Y.); samuel.ho.s.w@icloud.com (S.S.-W.H.); clairesophie1992@yahoo.com.sg (C.S.E.-S.L.); tweixun07@hotmail.com (W.-X.T.); phansy@nus.edu.sg (S.-Y.N.)

**Keywords:** *Leea indica*, HPLC-ESI-microTOF-Q-MS/MS, phenolics, dihydrochalcones

## Abstract

*Leea indica* (Vitaceae) is a Southeast Asian medicinal plant. In this study, an ethyl acetate fraction of *L. indica* leaves was studied for its phytoconstituents using high-performance liquid chromatography-electrospray ionization-mass spectrometry (HPLC-ESI-microTOF-Q-MS/MS) analysis. A total of 31 compounds of different classes, including benzoic acid derivatives, phenolics, flavonoids, catechins, dihydrochalcones, coumarins, megastigmanes, and oxylipins were identified using LC-MS/MS. Among them, six compounds including gallic acid, methyl gallate, (−)-epigallocatechin-3-*O*-gallate, myricetin-3-*O*-rhamnoside, quercetin-3-*O*-rhamnoside, and 4′,6′-dihydroxy-4-methoxydihydrochalcone 2′-*O*-β-d-glucopyranoside were isolated and identified by NMR analysis. The LC-MS/MS analysis led to the tentative identification of three novel dihydrochalcones namely 4′,6′-dihydroxy-4-methoxydihydrochalcone 2′-*O*-rutinoside, 4′,6′-dihydroxy-4-methoxydihydrochalcone 2′-*O*-glucosylpentoside and 4′,6′-dihydroxy-4-methoxydihydrochalcone 2′-*O*-(3″-*O*-galloyl)-β-d-glucopyranoside. The structural identification of novel dihydrochalcones was based on the basic skeleton of the isolated dihydrochalcone, 4′,6′-dihydroxy-4-methoxydihydrochalcone 2′-*O*-β-d-glucopyranoside and characteristic LC-MS/MS fragmentation patterns. This is the first comprehensive analysis for the identification of compounds from *L. indica* using LC-MS. A total 24 compounds including three new dihydrochalcones were identified for the first time from the genus *Leea*.

## 1. Introduction

*Leea indica* (Burm. f.) Merr. (Vitaceae), commonly known as Bandicoot berry, is an evergreen perennial shrub or a small tree of 2 to 16 m in height. It is distributed throughout Bangladesh, China, India, Malaysia, Singapore, North Australia, Thailand, and Vietnam [1,2,3]. Traditionally, *L. indica* is used as a remedy during pregnancy, for birth control, body pain, skin problems, and relief from dizziness [4,5]. *L. indica* is reported to possess various pharmacological activities, e.g., analgesic, anti-angiogenesis, anti-oxidant, anti-inflammatory, anti-microbial, anti-proliferative, hepatoprotective, sedative, and anxiolytic activities [3,5,6,7,8,9,10,11,12]. The plant contains different classes of compounds including phenolics, terpenoids, phthalic acid derivatives, and steroids [13,14,15]. Currently, there are very few reports available on the phytochemistry of *L. indica*. 

The objective of the present study was to isolate and identify chemical constituents from an ethyl acetate fraction of *L. indica* leaves. The comprehensive chemical identification was carried out by high performance liquid chromatography coupled to electrospray ionization and quadrupole time-of-flight mass spectrometry (HPLC-ESI-microTOF-Q-MS) analysis along with the isolation of compounds **1**, **5**, **10**, **14**, **18,** and **27** from ethyl acetate fraction. The structures of the isolated compounds were identified using NMR and MS analyses. A total of 31 compounds belonging to different classes including benzoic acid derivatives, flavonoids, coumarins, megastigmanes, catechins, dihydrochalcones, and oxylipins were identified. Here we report the identification of three novel dihydrochalcones along with 28 known compounds from the ethyl acetate fraction of *L. indica* leaves. In total, 24 compounds, including three novel dihydrochalcones, are reported for the first time in the genus *Leea*.

## 2. Results and Discussion

### 2.1. Isolation and Identification of Compounds

The methanolic extract of *L. indica* leaves was fractionated with hexane, dichloromethane and ethyl acetate. The dried yields were 0.005%, 0.027% and 1.32% respectively. Purification of the major organic ethyl acetate fraction by repeated column chromatography led to the isolation of compounds **1**, **5**, **10**, **14**, **18,** and **27**. The compounds were identified as gallic acid (**1**) [16], methyl gallate (**5**) [17], epigallocatechin-3-*O*-gallate (**10**) [18], myricetin-3-*O*-rhamnoside (**14**) [19], quercetin-3-*O*-rhamnoside (**18**), [19] and 4′,6′-dihydroxy-4-methoxydihydrochalcone 2′-*O*-β-d-glucopyranoside (**27**) [20] by comparing their analytical data (^1^H, ^13^C and 2D-NMR, and LC-MS) with those reported in the literature [16,17,18,19,20].

### 2.2. Identification of Dihydrochalcones by LC-ESI-MS/MS Analysis 

**Figure 1:**
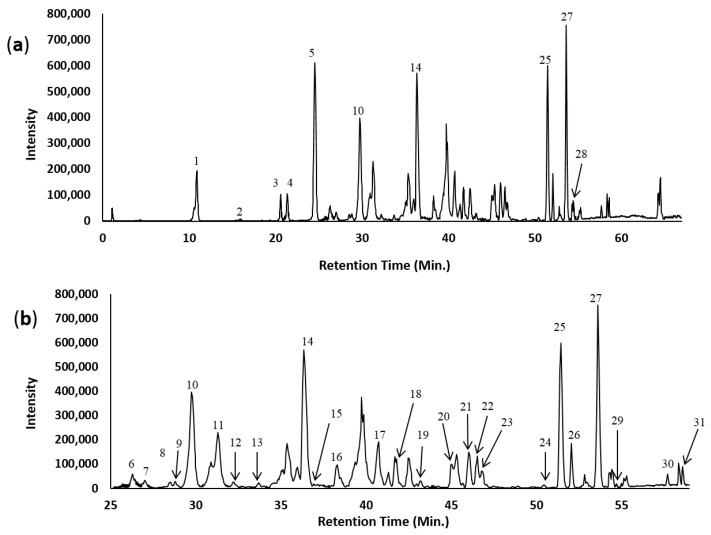
(**a**) Base peak chromatogram (BPC) of *L. indica* ethyl acetate fraction by HPLC-ESI-MS in negative ionization mode; (**b**) Expanded BPC. Peak labeling represents the compounds identified.

**Figure 2:**
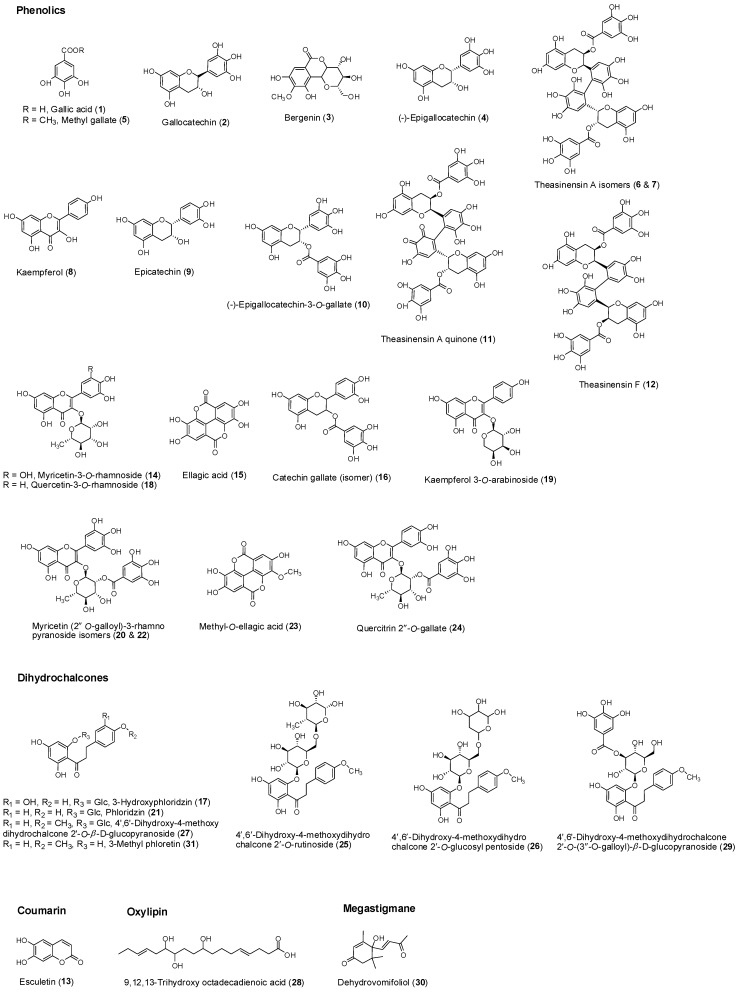
Structures of compounds identified in *L. indica* according to their chemical classes.

The ethyl acetate fraction of *L. indica* leaves was analyzed by the LC-ESI-MS/MS method. Figure 1 shows the base peak chromatogram (BPC) of the ethyl acetate fraction of *L. indica* leaves at 254 nm. Figure 2 shows the structures of the 31 compounds identified. In total, 31 compounds were identified of which ten compounds (**1**, **4**, **5**, **8**, **9**, **10**, **14**, **15**, **18** and **21**) were verified by comparison with reference standards. Seven compounds were tentatively identified as dihydrochalcone derivatives: 3-hydroxyphloridzin **17**, phloridzin **21**, 4′,6′-dihydroxy-4-methoxydihydrochalcone 2′-*O*-rutinoside **25** (*m*/*z* 595), 4′,6′-dihydroxy-4-methoxydihydrochalcone 2′-*O*-glucosyl pentoside **26** (*m*/*z* 581), 4′,6′-dihydroxy-4-methoxydihydrochalcone 2′-*O*-β-d-glucopyranoside **27**, 4′,6′-dihydroxy-4-methoxydihydrochalcone 2′-*O*-(6″-*O*-galloyl)-β-d-glucopyranoside **29** (*m*/*z* 601) and 2′,4′,6′-trihydroxy-4-methoxydihydrochalcone (3-methylphloretin) **31**. Compounds **25**, **26** and **29** are reported for the first time. While dihydrochalcone phloridzin has been previously reported in *L. indica* [13], the other six dihydrochalcone derivatives have not been previously reported in the same plant species. The observed MS peaks including retention time, observed mass, calculated mass, molecular formula, ppm error, and MS/MS data are presented in Table 1.

The structural identification of three new dihydrochalcones **25**, **26** and **29** was based on the relevance of the LC-MS/MS fragmentation patterns with the isolated compound 4′,6′-dihydroxy-4-methoxy dihydrochalcone 2′-*O*-β-d-glucopyranoside **27**. The MS/MS spectra of compounds **25**, **26**, **27** and **29,** showed a common base ion peak at *m*/*z* 287 for 2′,4′,6′-trihydroxy-4-methoxydihydrochalcone, which is a characteristic ion formed by the loss of glycoside(s) and/or galloyl glycoside moieties.

In LC-MS spectra, peaks **25**, **26** and **29** eluted at retention times (RT) 51.4, 52.1 and 55.0 min, and showed precursor ions [M − H]^−^ at *m*/*z* 595.2031, 581.1889 and 601.1595, respectively. Peaks **25** (*m*/*z* 595) and **26** (*m*/*z* 581) showed a mass difference of 146 Da (rhamnose) and 132 Da (arabinose/xylose) respectively compared to the isolated dihydrochalcone **27** (*m*/*z* 449). Also, peak **26** (*m*/*z* 581) was found to be 14 Da lighter than peak **25** (*m*/*z* 595), indicating the presence of a pentose sugar. In agreement with mass analysis data, peaks **25** and **26** were tentatively characterized as 4′,6′-dihydroxy-4-methoxydihydrochalcone 2′-*O*-rutinoside (*m*/*z* 595) and 4′,6′-dihydroxy-4-methoxydihydrochalcone 2′-*O*-glucosylpentoside (*m*/*z* 581) respectively.

**Scheme 1:**
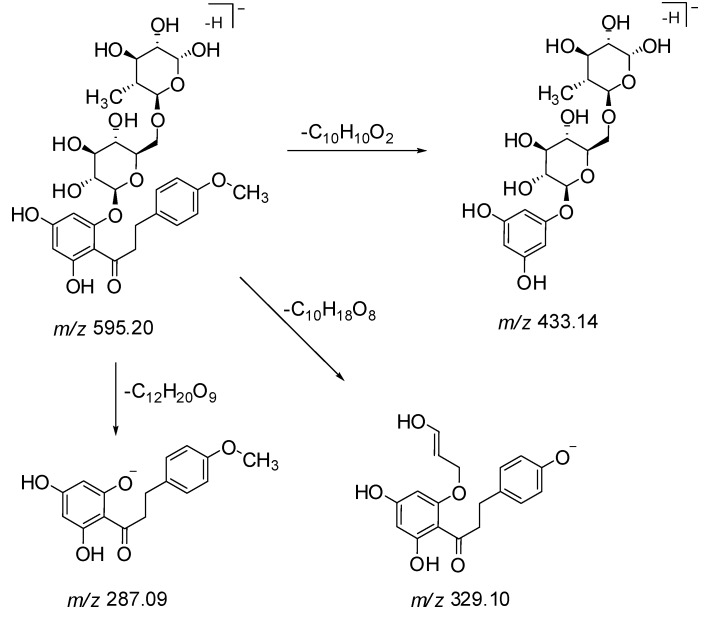
Proposed MS/MS fragmentation of compound **25**.

Peak **25** displayed a molecular ion [M − H]^−^ at *m*/*z* 595.2031 (C_28_H_36_O_14_) and fragment ions at *m*/*z* 433, 329 and 287 (Scheme 1 and Figure S1). In the MS/MS spectrum, a characteristic fragment ion at *m*/*z* 287 as base peak suggested that this compound corresponded to a 2′,4′,6′-trihydroxy-4-methoxydihydrochalcone linked to a rutinose moiety, where the neutral loss of 308 Da is characteristic of the loss of a rutinose moiety [21]. The fragments at *m*/*z* 433 [M − C_10_H_10_O_2_ − H]^−^ and 329 [M − C_10_H_18_O_8_ − H]^−^ were obtained by the cleavage of the C-C bond of chalcone and sugar moiety respectively (Scheme 1). The fragment ion at *m*/*z* 329 [M − H − C_9_H_13_O_7_ − H_2_O − CH_3_]^−^ was obtained by the cleavage of a glucose moiety, with the loss of a water molecule and further by losing a methyl group. Based on these deductions, peak **25** was tentatively identified as 4′,6′-dihydroxy-4-methoxydihydrochalcone 2′-*O*-rutinoside, a new dihydrochalcone.

**Scheme 2:**
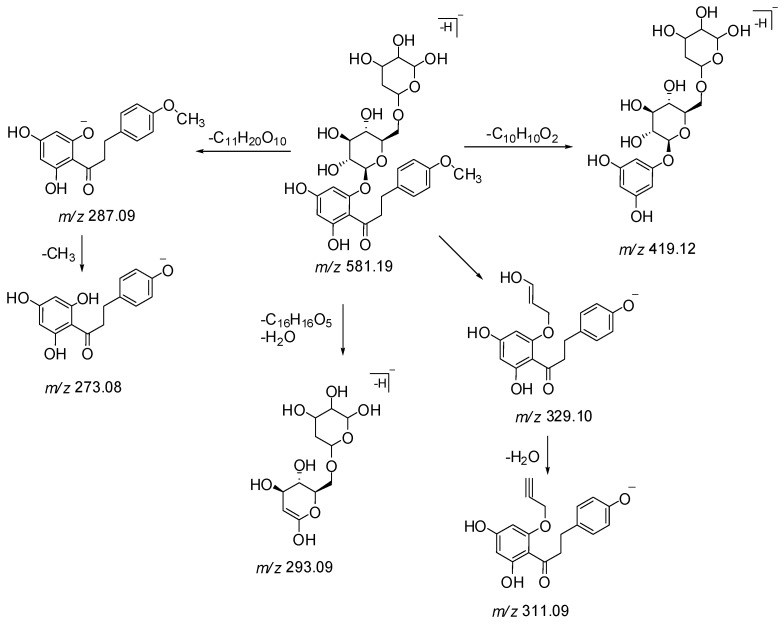
Proposed MS/MS fragmentation of compound **26**.

**Scheme 3:**
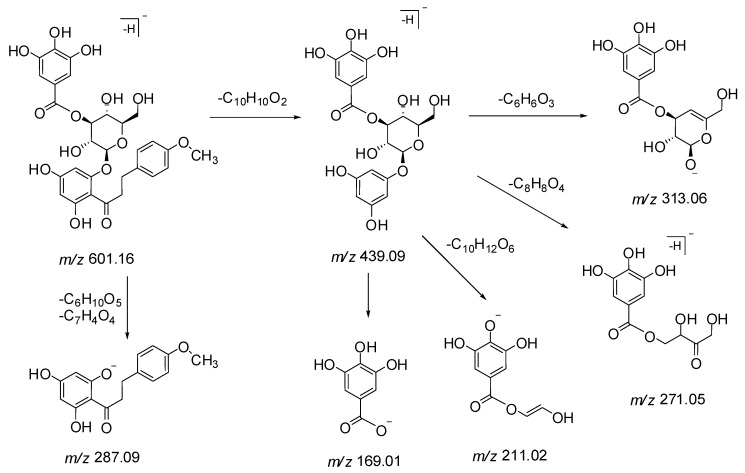
Proposed MS/MS fragmentation of compound **29**.

Peak **26** exhibited a precursor ion [M − H]^−^ at *m*/*z* 581.1889 (C_27_H_34_O_14_) and fragment ions at *m*/*z* 419, 311, 293, and 243 (Scheme 2 and Figure S2). The MS/MS spectrum showed product ion at *m*/*z* 287 (C_16_H_16_O_5_) [M − H − 162 Da − 132 Da]^−^ as base peak by the loss of a glucosylpentoside moiety, suggesting to possess a basic skeleton of isolated dihydrochalcone **27**. The cleavage of a C-C bond gave a fragment ion at *m*/*z* 419 due to the loss of a C_10_H_10_O_2_ moiety. The neutral loss of 312 Da showed the presence of a glucosyl pentoside moiety, losing a molecule of water to generate a product ion at *m*/*z* 293 (Scheme 3). Therefore, compound **26** was plausibly identified as 4′,6′-dihydroxy-4-methoxydihydrochalcone 2′-*O*-glucosylpentoside and found as first occurrence in nature. 

Peak **29** showed a precursor ion [M − H]^−^ at *m*/*z* 601.1595 (C_29_H_30_O_14_) and fragment ions at *m*/*z* 439, 313, 287, 271, 211, and 169 in the MS/MS spectrum. A base ion peak at *m*/*z* 287 [M − C_6_H_10_O_5_ − C_7_H_4_O_4_ − H]^−^ was observed due to the loss of glucose (162 Da) and galloyl (153 Da) moieties. Fragment ions at *m*/*z* 169 and *m*/*z* 313 indicate the presence of a galloyl and a galloylglucose moiety respectively. Monogalloylglucose can exist as five possible isomers namely, 1-*O*-galloylglucose, 2-*O*-galloylglucose, 3-*O*-galloylglucose, 4-*O*-galloylglucose, and 6-*O*-galloylglucose [22]. The characteristic fragment ions at *m*/*z* 271 and 211 suggest that the substitution of the galloyl group could be at the C-3 position of the glucose moiety (Scheme 3 and Figure S3). Product ion detected at *m*/*z* 439 suggested the cleavage of the C-C bond (loss of C_10_H_10_O_2_ moiety) in the MS/MS spectrum. Thus, the compound corresponding to peak **29** was tentatively identified as a new dihydrochalcone 4′,6′-dihydroxy-4-methoxydihydrochalcone 2′-*O*-(3″-*O*-galloyl)-β-d-glucopyranoside. It is also a gallic acid derivative of the isolated dihydrochalcone **27**.

The isolated dihydrochalcone **27** exhibited a precursor ion [M − H]^−^ at *m*/*z* 449.1452 (C_22_H_26_O_10_). The MS/MS spectrum showed product ions at *m*/*z* 287 [M − C_6_H_10_O_5_ −H]^−^ and 273 [M − C_6_H_10_O_5_ − CH_3_ − H]^−^ due to the loss of glucose (162 Da) and methyl groups (15 Da) (Figure S4). Compound **27** was isolated and identified as 4′,6′-dihydroxy-4-methoxydihydrochalcone 2′-*O*-β-d-glucopyranoside. 

The LC-MS fragmentation patterns of the three novel dihydrochalcones (**25**, **26** and **29**) were compared to the isolated dihydrochalcone (**27**), and we noted that the observed HR-MS data were in good agreement with the calculated masses. Further isolation of the peaks **25**, **26** and **29** and spectroscopic analyses would be required to unambiguously confirm the proposed structures of these dihydrochalcones.

## 3. Materials and Methods 

### 3.1. Plant Materials 

Fresh ground leaves of *L. indica* were collected in Singapore. A voucher specimen (no. LI-0109) was deposited at the herbarium of the National University of Singapore (NUS) Medicinal Plant Research Group.

### 3.2. Chemicals and Reagents

Standards gallic acid, methyl gallate, myricitrin, quercitrin, epigallocatechin-3-*O*-gallate, ellagic acid, epicatechin, and kaempferol were purchased from Sigma-Aldrich (St. Louis, MO, USA). Phloridzin and epigallocatechin were purchased from TCI Co. Ltd. (Tokyo, Japan). LC-MS grade solvents (acetonitrile, methanol and formic acid) were purchased from MERCK (Darmstadt, Germany) and water used in LC analysis was obtained using Milli-Q advanced system (Millipore, Milford, MA, USA).

### 3.3. Extraction and Isolation

The fresh ground leaves of *L. indica* (2.8 kg) were macerated with 70% *v*/*v* MeOH at room temperature. The extract was filtered and concentrated under vacuum, yielding a crude methanolic extract. The dried methanolic extract was dissolved in water and partitioned with different solvents, concentrated under vacuum to give hexane (0.005%), dichloromethane (0.027%) and ethyl acetate (1.32%) fractions. 

The ethyl acetate fraction (37.0 g) was chromatographed over silica gel using 25% EtOAc–hexane as eluent, yielding a white solid, which was recrystallized in CHCl_3_-MeOH as white needles of methyl gallate (60 mg). Fractions obtained from repeated silica gel column chromatography of EtOAc fraction using 6–10% MeOH-CHCl_3_ as eluent were further purified by Sephadex (LH-20) and reversed phase cartridge yielding two compounds gallic acid (140 mg) and 4′,6′-dihydroxy-4-methoxydihydrochalcone 2′-*O*-β-d-glucopyranoside (12 mg). The estimated concentration of gallic acid in the fresh leaves was 0.005–0.011% *w*/*w*. Pooled fractions obtained from silica gel column chromatography of the EtOAc fraction using 10–20% MeOH-CHCl_3_ were further subjected to Sephadex (LH-20) column chromatography. At an eluent concentration of 50% MeOH-water, a mixture of two compounds was obtained. It was further purified by silica gel column chromatography eluting with 8% MeOH-CHCl_3_ and 10–12% MeOH-CHCl_3_ to yield quercetin-3-*O*-rhamnoside (5 mg) and myricetin-3-*O*-rhamnoside (650 mg) respectively. Epigallocatechin-3-*O*-gallate (64 mg) was obtained from the silica gel column chromatography using 2–5% methanol in dichloromethane. The structures of isolated compounds **1**, **5**, **10**, **14**, **18,** and **27** were confirmed by NMR and LC-MS analyses. 

### 3.4. General Information

NMR spectra were recorded on a Bruker Avance-400 Spectrometer (Fallanden, Switzerland), ^1^H at 400 MHz and ^13^C at 100 MHz in deuterated solvents using tetramethylsilane (TMS) as an internal reference. Deuterated solvents, methanol-*d*_4_ and dimethyl sulfoxide-*d*_6_ for NMR were purchased from Sigma-Aldrich (USA).

Silica-gel (60–120, 100–200, 70–230 mesh; Merck, Germany), Sephadex LH-20 (Sigma, Uppsala, Sweden) and reversed phase C18 (77.9 μm) cartridge column from Waters (Ireland) were used for chromatographic separation. Thin layer chromatography was performed on pre-coated Si-gel 60 F_254_ plates (Merck, Germany) using a visualizing reagent. 

The LC-MS analysis was carried out using a Dionex Ultimate 3000 VWD system coupled with a VWD and a micro-TOF-Q mass detector (Bruker Daltonics Inc., Billerica, MA, USA). Chromatographic separation was performed on an RP-C_18_ column (3.0 × 150 mm; particle size 2.7 μM; Agilent Poroshell 120, New Castle, DE, USA), operated at 25 °C. Analysis was carried out using a gradient elution program of 0.1% formic acid in water (A) and 0.1% formic acid in acetonitrile (B) as a mobile phase at a flow rate of 0.5 mL/min. The following gradient system was used: 0–45 min, 5–30% B; 45–60 min 30–100% B and 60–65 min 100% B. UV detection was performed by scanning the samples at 210, 254, 280, and 360 nm. Electrospray ionization mass spectra (ESI-MS) were recorded in negative ionization mode. The mass range of *m*/*z* 50–2000 was scanned. For MS/MS analysis, collision energies were set automatically. 

## 4. Conclusions

This study presents the comprehensive identification of chemical constituents of an ethyl acetate fraction of *L. indica* leaves using HPLC-ESI-microTOF-Q-MS/MS analysis. Here we identified 31 compounds, among them six phenolic compounds were isolated by column chromatography. Three novel dihydrochalcones derivatives were tentatively identified as 4′,6′-dihydroxy-4-methoxydihydrochalcone 2′-*O*-rutinoside, 4′,6′-dihydroxy-4-methoxydihydro chalcone 2′-*O*-glucosylpentoside and 4′,6′-dihydroxy-4-methoxydihydrochalcone 2′-*O*-(3″-*O*-galloyl)-β-D-glucopyranoside. A total of 24 compounds are reported for the first time in the genus *Leea*. Our results indicated that *L. indica* is a good source of diverse phenolic contents including phenolic acids (gallic acid and methyl gallate), polyphenolic (ellagic acid), flavan-3-ols (gallocatechin, epigallocatechin and epigallocatechin-3-*O*-gallate), flavonoids/flavonoid glycosides (kaempferol, quercitrin, myricitrin), dihydrochalcones (phloridzin and its derivatives), and dimeric catechins (theasinensin A dimers and theasinensin F). The wide range of potential bioactive compounds supports the diverse pharmacological activities of *L. indica*. Further research to identify and develop useful therapeutics and health supplements from *L. indica* is warranted. 

## Figures and Tables

**Table 1:** Identification of compounds from ethyl acetate fraction of *L. indica* by HPLC-ESI-microTOF-Q-MS/MS at 254 nm in negative ionization mode.

Peak no.	RT (min)	Observed [M − H]^−^	Calculated [M − H]^−^	Error (ppm)	Molecular Formula	Fragment Ions (*m/z*)	Identified Compound
1	10.9	169.0146	169.0142	−2.2	C_7_H_6_O_5_	125.0444	Gallic acid
2	15.9	305.0668	305.0667	−0.4	C_15_H_14_O_7_	261.0623, 219.0682, 179.0279, 167.0371, 165.0179, 151.1024	Gallocatechin ^†^
3	20.6	327.0726	327.0722	−1.4	C_14_H_16_O_9_	312.0487, 234.0173, 207.0298, 206.0222, 192.0079	Bergenin
4	21.4	305.0668	305.0667	−0.4	C_15_H_14_O_7_	287.059, 261.076, 219.0694, 221.0473, 179.0362, 167.0387, 165.0199	Epigallocatechin ^†^
5	24.5	183.0304	183.0299	−2.7	C_8_H_8_O_5_	169.0107	Methyl gallate ^†^
6	26.3	913.1455	913.1469	1.6	C_44_H_34_O_22_	761.1369, 743.1264, 609.1287, 591.1153, 573.1038, 447.0733, 423.0709, 285.0410, 169.0143	Theasinensin A (isomer 1) ^†^
7	27.0	913.1471	913.1469	−0.2	C_44_H_34_O_22_	761.131, 743.1255, 609.1205, 591.1148, 573.1104, 447.0721, 423.0752, 285.0422, 169.0178	Theasinensin A (isomer 2) ^†^
8	28.5	285.0399	285.0405	2.1	C_15_H_10_O_6_	243.0291, 217.0528, 199.0420, 175.047	Kaempferol
9	28.8	289.0721	289.0718	−1.0	C_15_H_14_O_6_	221.0795, 203.0724, 175.0323	Epicatechin
10	29.8	457.0784	457.0776	−1.6	C_22_H_18_O_11_	305.0660, 261.0803, 219.0637, 169.0142	Epigallocatechin-3-*O*-gallate ^†^
11	31.0	911.1315	911.1312	−0.2	C_44_H_32_O_22_	759.1258, 741.1135, 589.1027, 571.0861, 441.0556, 423.0727, 305.0618, 301.0453, 285.0431, 169.0135	Theasinensin A quinone ^†^
12	32.2	897.1515	897.1520	0.5	C_44_H_34_O_21_	745.1526, 727.1485, 575.1195, 557.1, 449.0938, 423.0693, 287.0576, 269.0482, 169.0127	Theasinensin F ^†^
13	33.7	177.0191	177.0193	1.2	C_9_H_6_O_4_	148.9428, 132.9003, 105.9031	Esculetin ^†^
14	36.4	463.0886	463.0882	−0.8	C_21_H_20_O_12_	317.029, 316.0226, 287.0199, 271.0247, 179.0012, 135.8248	Myricetin 3-*O*-rhamnoside (myricitrin)
15	36.9	300.9989	300.9990	0.2	C_14_H_6_O_8_	283.9927, 245.0151, 229.0091, 201.0309, 200.0171, 173.0194	Ellagic acid ^†^
16	38.3	441.0831	441.0827	−0.9	C_22_H_18_O_10_	289.0701, 271.06, 245.9752, 169.0132	Catechin gallate (isomer) ^†^
17	41.2	451.1254	451.1246	−1.7	C_21_H_24_O_11_	289.0724, 271.1548, 167.0353	3-Hydroxyphloridzin ^†^
18	41.7	447.0931	447.0933	0.4	C_21_H_20_O_11_	301.0325, 300.0271, 255.0296, 179.0009	Quercetin 3-*O*-rhamnoside (Quercitrin)
19	43.2	417.0833	417.0827	−0.6	C_20_H_18_O_10_	284.0316, 257.0446, 255.0304, 227.0339	Kaempferol 3-*O*-arabinoside ^†^
20	45.0	615.1001	615.0992	−1.5	C_28_H_24_O_16_	463.0903, 317.0319, 297.0616, 178.9989, 169.0188	Myricetin-*O*-(*O*-galloyl)-3-rhamnopyranoside (isomer 1) ^†^
21	46.0	435.1299	435.1297	−0.5	C_21_H_24_O_10_	273.0758, 167.0349	Phloridzin
22	46.5	615.0988	615.0992	0.6	C_28_H_24_O_16_	463.0817, 317.0332, 297.0677, 178.9976, 169.011	Myricetin-*O*-(*O*-galloyl)-3-rhamnopyranoside (isomer 2) ^†^
23	46.8	315.0146	315.0146	0.1	C_15_H_8_O_8_	299.9902, 270.9912, 243.9987, 151.0037	Methyl-*O*-ellagic acid ^†^
24	50.4	599.1048	599.1042	−1.0	C_28_H_24_O_15_	447.0893, 301.0369, 169.0125, 151.8637	Quercitrin 2″-*O*-gallate ^†^
25	51.4	595.2031	595.2032	0.2	C_28_H_36_O_14_	433.1347, 329.1078, 308.2508, 287.0929, 167.0376	4′,6′-Dihydroxy-4-methoxy dihydrochalcone 2′-*O*-rutinoside ^†^
26	52.1	581.1889	581.1876	−0.5	C_27_H_34_O_14_	419.1210, 329.102, 311.0951, 293.0907, 287.0926, 273.0953, 243.1026, 167.0355	4′,6′-Dihydroxy-4-methoxy dihydrochalcone 2′-*O*-glucosylpentoside ^†^
27	53.6	449.1452	449.1453	0.4	C_22_H_26_O_10_	329.1080, 287.0921, 273.0744, 272.0683, 243.1032, 181.017, 167.0298, 166.0275, 151.0067	4′,6′-Dihydroxy-4-methoxy dihydrochalcone 2′-*O*-β-d-glucopyranoside ^†^
28	54.5	327.2171	327.2177	1.8	C_18_H_32_O_5_	309.2164, 298.9867, 291.1998, 239.1283, 229.1447, 211.1327, 183.0131, 171.103	9,12,13-Trihydroxy octadecadienoic acid ^†^
29	55.0	601.1595	601.1563	−5.3	C_29_H_30_O_14_	439.0901, 329.1098, 313.0559, 287.0914, 271.0502, 243.1106, 211.0199, 169.0167	4′,6′-Dihydroxy-4-methoxy dihydrochalcone 2′-*O*-(3″-*O*-galloyl)-β-d-glucopyranoside ^†^
30	57.7	221.1186	221.1183	−1.4	C_13_H_18_O_3_	149.0978	Dehydrovomifoliol ^†^
31	58.6	287.0926	287.0925	−0.2	C_16_H_16_O_5_	243.1034, 167.037, 151.0043	2′,4′,6′-Trihydroxy-4-methoxy dihydrochalcone (3-Methylphloretin) ^†^

^†^ Compounds identified for the first time in the genus *Leea*.

## Supplementary Materials

Supplementary materials are available online. Figure S1: MS^2^ spectrum and proposed fragmentation pattern of compound **25**; Figure S2: MS^2^ spectrum and proposed fragmentation pattern of compound **26**; Figure S3: MS^2^ spectrum and proposed fragmentation pattern **29**; Figure S4: MS^2^ spectrum and proposed fragmentation pattern of compound **27**.
