# Supplementary material for: Identification of Phytoconstituents in Leea indica (Burm. F.) Merr. Leaves by High Performance Liquid Chromatography Micro Time-of-Flight Mass Spectrometry
Source: Molecules. 2019 Feb 16;24(4):714. doi: 10.3390/molecules24040714 (PMC6412998; doi:10.3390/molecules24040714)
Supplement: Supplementary file 1 [file molecules-24-00714-s001.pdf]

Supporting information

Identification of Phytoconstituents of *Leea indica* (Burm. F.) Merr. Leaves by High Performance Liquid Chromatography Micro Time-of-Flight Mass Spectrometry

Deepika Singh*, Yin-Yin Siew, Teck-Ian Chong, Hui-Chuing Yew, Samuel Shan-Wei Ho, Claire Sophie En-Shen Lim, Wei-Xun Tan, Soek-Ying Neo, Hwee-Ling Koh*

Department of Pharmacy, Faculty of Science, National University of Singapore, 18 Science Drive 4, Singapore 117543, Singapore

*Correspondence: phads@nus.edu.sg; phakohhl@nus.edu.sg; Tel.: +65-65163120 (H.-L.K.)

Table of contents

MS^2 spectrum and proposed fragmentation pattern of compound **25** (FigureS1)................S2

MS^2 spectrum and proposed fragmentation pattern of compound **26** (Figure S2)...............S2

MS^2 spectrum and proposed fragmentation pattern of compound **29** (Figure S3)...............S3

MS^2 spectrum and proposed fragmentation pattern of compound **27** (Figure S4)...............S3

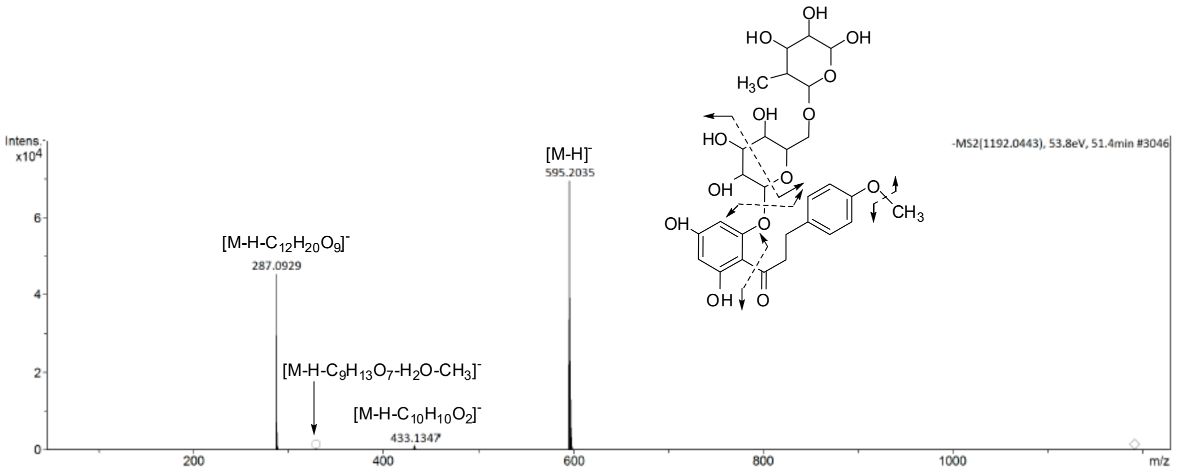

Figure S1. MS^2 spectrum and proposed fragmentation pattern of compound **25**.

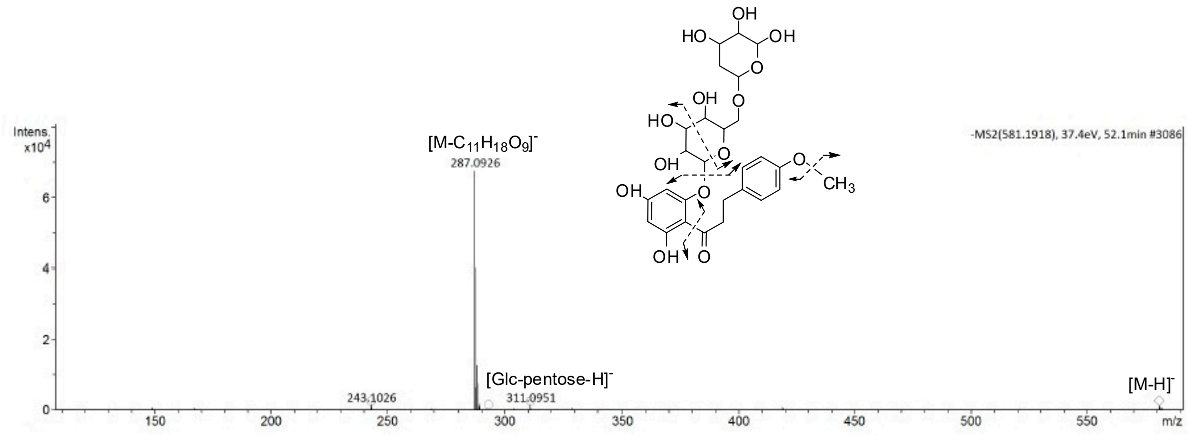

Figure S2. MS^2 spectrum and proposed fragmentation pattern of compound **26**.

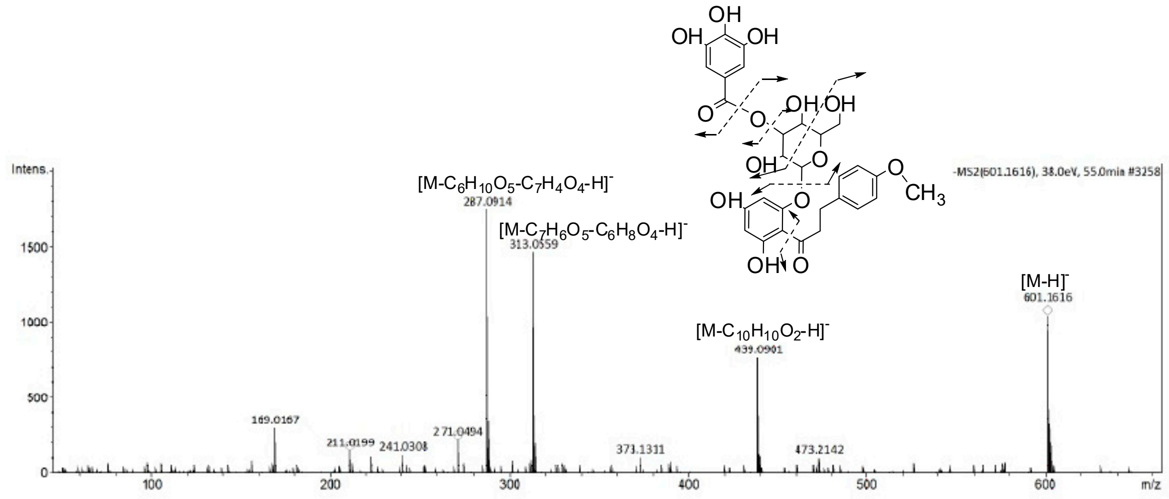

Figure S3. MS^2 spectrum and proposed fragmentation pattern of compound **29**.

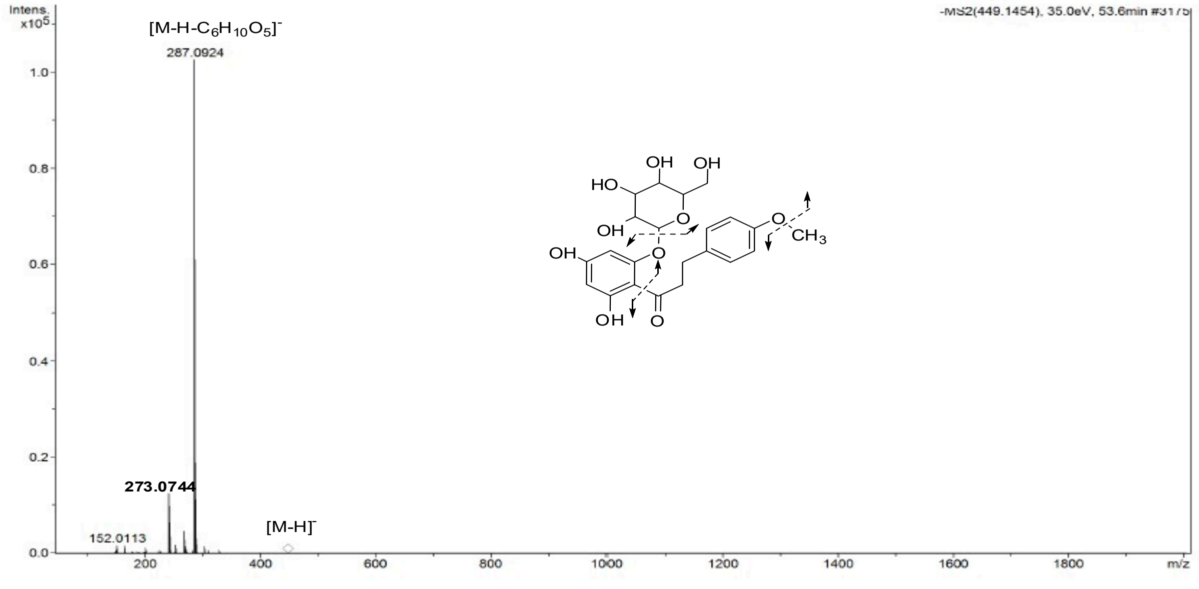

Figure S4. MS^2 spectrum and proposed fragmentation pattern of compound **27**.
